# Evaluation of a Deep Learning-Based Index for Prognosis of a Vessel’s Propeller-Hull Degradation

**DOI:** 10.3390/s23218956

**Published:** 2023-11-03

**Authors:** Christos Spandonidis, Dimitrios Paraskevopoulos

**Affiliations:** Prisma Electronics S.A., Research, and Development, 87 Democratias Avenue, 68132 Alexandroupolis, Greece; d.paraskevopoulos@prismael.com

**Keywords:** maintenance, prognosis, KPIs, Artificial Neural Networks, ISO-19030, propeller-hull

## Abstract

Vessels frequently encounter challenging marine conditions that expose the propeller-hull to corrosive water and marine fouling. These challenges necessitate innovative approaches to optimize propeller-hull performance. This study aims to assess a method for predicting propeller-hull degradation. The proposed solution revolves around an innovative Key Performance Indicator (KPI) based on Artificial Neural Networks (ANNs). Our objective is to validate the findings; thus, a thorough comparison is conducted between the proposed method and the baseline solution derived from the ISO-19030. Emphasis is placed on determining the optimal parameters for computing the KPI, which involves applying various features, filters, and pre-processing techniques. The proposed method is tested on real data collected by an Internet of Things (IoT) system installed in different types of vessels. Four distinct experiments with ANNs are conducted. Results demonstrate that the ANN-based indicator offers greater accuracy in predicting propeller-hull degradation compared to the baseline method. Additionally, it is demonstrated that selecting a diverse set of features and implementing consistent filtering and preprocessing techniques enhance the performance of the traditional indicator. The utilization of Deep Learning (DL) in the maritime industry is of great significance, as it enables a comprehensive and dynamic assessment of predictive maintenance of the propeller-hull. The DL index method holds potential for diverse maintenance applications, providing a holistic platform with anticipated environmental and financial benefits.

## 1. Introduction

Throughout their life cycle, vessels are exposed to harsh marine environments where seawater acts as an electrolyte, triggering the electrochemical process of corrosion. This corrosion leads to long-term damage in vessels, reducing the mechanical properties of the propeller-hull. Recent studies have highlighted another challenge in the form of marine fouling, caused by the abundance of microorganisms in the water. This fouling increases the surface roughness of the vessel [1,2]. Both corrosion and marine fouling degrade the performance of the propeller-hull, resulting in an increased power requirement [3]. Research indicates that the proper functioning of the propeller-hull has a significant impact on the overall performance of the vessel. This includes reducing fuel consumption and air pollutant emissions, such as carbon dioxide (CO_2_), in the atmosphere [4,5,6]. By monitoring, sustaining, and improving the propeller-hull performance, costs can be reduced by 50% and air pollutant emissions by 30%, leading to a growth in international trade [7]. To achieve these outcomes, predictive maintenance plays a crucial role by allowing vessel operators to proactively address potential performance issues and failures. Predictive maintenance aims to determine the condition of critical systems in the vessel and identify the presence and severity of faults. Fault detection is the initial step in a data-driven predictive maintenance system, with the goal of identifying faults. Fault diagnosis involves identifying the specific type of fault, while prognosis focuses on predicting when the failure may occur in the system. Various techniques have emerged for monitoring the propeller-hull condition of a ship using Key Performance Indicators (KPIs). These techniques can be broadly categorized into traditional and enhanced data-driven approaches [8,9].

The utilization of traditional techniques in the maritime industry is founded on the ISO-19030, a collection of standards developed by the International Organization for Standardization. The ISO-19030 serves as a means for the maritime industry to reduce its environmental impact and enhance operational efficiency by providing a standard for estimating propeller-hull performance. The fundamental concept of the ISO-19030 involves the computation of a performance indicator over a specific time frame, which is defined as the percentage difference between the anticipated value of the ship’s speed and the measured value at the same shaft power. In the study conducted by Papageorgiou et al. [10], the ISO-19030 was utilized to monitor the condition of the hull and propeller. Shaw and Lin [8] employed an ISO-19030-based KPI to monitor energy efficiency by utilizing measurements obtained from various sources. Koboevic et al. [11] relied on data trends to monitor hull and propeller fouling through the ISO-19030 to determine the optimal dry-docking period. In previous works, Themelis et al. [12] presented a platform enriched with several KPIs targeting various aspects of vessel performance, including a KPI that relied on the ISO-19030.

In the context of the fourth industrial revolution, Deep Learning (DL) has emerged as a transformative force that leverages the collected sensor data. To estimate the corrosion and coating defect of coal handling and preparation plants, Yu et al. [13] proposed an innovative machine vision-based approach that comprises an ensemble of Convolutional Neural Network (CNN) and improved Dempster–Shafer theory-based data fusion. Additionally, Yu et al. [14] presented a novel hybrid framework of optimized DL models combined with multi-sensor fusion for the condition diagnosis of concrete arch beams. Furthermore, the authors in [15] emphasized the accuracy of 2D-CNN models for analyzing images from low-frequency sensor data.

Recent advancements in ship energy efficiency measurement techniques have introduced novel approaches that utilize Machine Learning and DL algorithms [9]. For instance, Corradu et al. employed Monte-Carlo simulations and DL implementations to estimate the energy performance indicator [9,16]. In the latter case, the results were compared to the baseline KPI computed using the ISO-19030 standard. In a study conducted by Laurie et al. [17], five Machine Learning models were evaluated for predicting shaft power and identifying performance deterioration caused by fouling. The authors concluded that the Random Forest model exhibited the greatest effectiveness in this regard. Gupta et al. experimented with various Machine Learning methods to estimate the hydrodynamic performance of a ship using recorded in-service data [18]. Interestingly, the authors emphasized that simple interpretable models can outperform complex black-box models when domain knowledge is incorporated. Mittendorf et al. generated synthetic monitoring data and trained Neural Networks to estimate the required shaft power for hull and propeller performance changes [19]. The study also demonstrated the value of freezing layers during incremental learning. Uzun et al. proposed a time-dependent biofouling growth model and compared the predictions with ship performance reports provided by vessel operators [20]. Lastly, Nowruzi presented an equation for predicting the hydrodynamic performance of stepped planing hulls based on the characteristics of designed Artificial Neural Networks (ANNs) [21].

Previous research conducted by Theodoropoulos et al. [22] and Spandonidis et al. [23] has explored the applications of ANNs and Recurrent Neural Networks (RNNs). Building upon this work, Theodoropoulos et al. [24] developed a KPI to evaluate the propeller-hull performance of a vessel using ANNs for diagnosing degradation. However, these studies were limited to the diagnosis of degradation, neglecting the crucial aspect of predicting and preventing failures through prognosis, which is an essential component of asset management. The present study aims to extend the scope of previous research by focusing on the reliability of the proposed KPI for forecasting degradation. The study has two objectives: first, to ensure the accuracy of the results by conducting a thorough comparison between the proposed KPI and the baseline KPI derived from ISO-19030 standards; second, to determine the optimal parameters for calculating the innovative KPI by applying several features, filters, and pre-processing techniques. To verify the results, the proposed method is implemented for different types of vessels.

The novelty of this study in predicting propeller-hull degradation is significant for the maritime industry and forms an integral component of a broader predictive maintenance solution. To the best of our knowledge, no other proposed methods forecast propeller-hull degradation based on KPIs. The remainder of the paper is organized as follows: Section 2 elaborates on the technical details of the proposed method for prognosis. Section 3 presents a brief description of the measurements obtained to conduct the experiments. Section 4 presents the results that emerged from employing the proposed method, while Section 5 compares its prognostic capabilities with a baseline method. Finally, Section 6 draws the final conclusions.

## 2. Method Description

The proposed prognostic methodology involves projecting the regression line of a novel KPI derived from propeller-hull performance data using ANNs. Prior to the application of ANNs, data preprocessing is a crucial step in the proposed methodology. This section provides a comprehensive overview of all the fundamental components of the proposed approach.

### 2.1. Data Preprocessing

**Figure 1:**
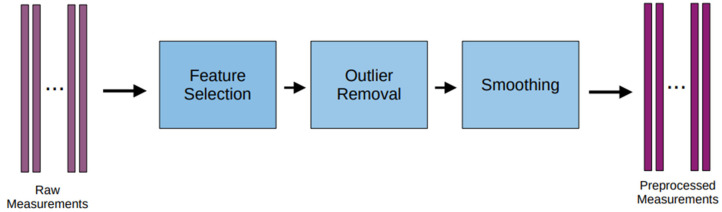
Flowchart of the preprocessing pipeline.

The measurements undergo preprocessing through the data-preprocessing pipeline depicted in Figure 1, as presented by Theodoropoulos et al. [24]. The obtained measurements are subjected to preprocessing by employing an iterative feature selection technique, which aims to identify the most influential features pertaining to the primary feature. Subsequently, the outliers are eliminated, and a smoothing process is applied.

Considering the outliers removal technique, the extreme values that deviate from the other observations in the dataset are identified and removed. More specifically, a parameter that coincides with the main feature is initially selected, and a data point is considered an outlier if the following condition is met:

(1) $$\left|data-\mu \right|\geq k*\sigma ,$$

where data, μ, σ, and k denote the data point, the mean, and the standard deviation of the secondary feature and a constant that controls the intensity of the outlier’s removal.

Furthermore, the measurements pass through the Simple Moving Average (SMA) algorithm. The SMA aims to compute the unweighted average of the last *n* samples of the window. Mathematically, the SMA is defined as:

(2) $$SMA=\frac{1}{n}\sum_{i=1}^{n}x_{i}\;\;,$$

where n and $x_{i}$ denote the number of samples and the $i^{th}$ observation in the time window, respectively. The SMA algorithm is applied due to its ability to remove any fluctuations while also capturing patterns present in the filtered dataset.

Considering the feature selection technique, the goal is to identify the most important features. Each feature undergoes an evaluation process for its relevance to the chosen target feature using a Random Forest regressor. In order to accomplish this, a level of significance is selected, and once the regressor is trained, the *p*-value of each feature is computed. If a feature’s *p*-value exceeds the significance level, it is removed from the dataset.

### 2.2. Artificial Neural Newtorks

**Figure 2:**
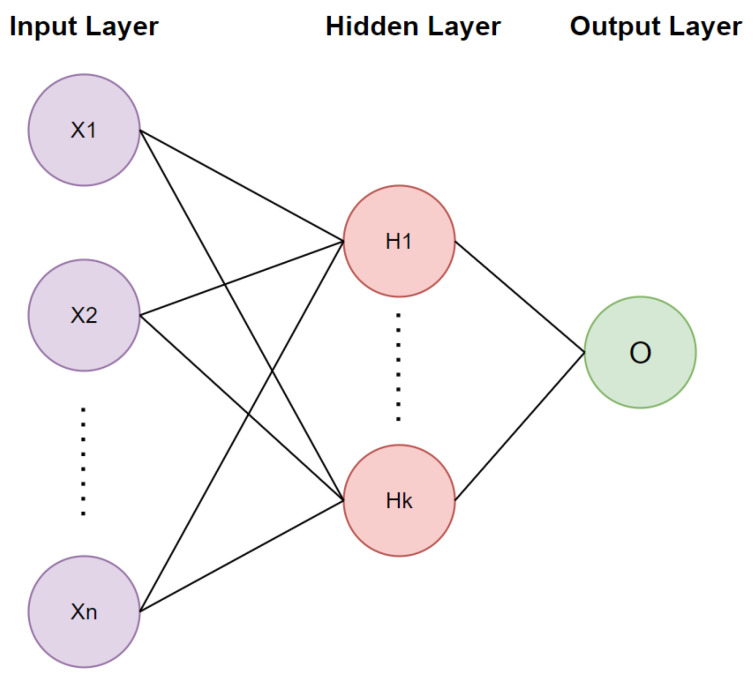
Architecture of Artificial Neural Networks (ANNs).

ANNs aim to model complex relationships and patterns within data by simulating the structure of the human brain, and they comprise input neurons/nodes that correspond to the input data, hidden layers with nodes, and output nodes. Each hidden layer connects its nodes with the nodes of other hidden layers, while the output may consist of one or several nodes, depending on the task of the ANN. In this case, ANNs are utilized in a regression task, and therefore the number of output nodes is equal to one. Weights are assigned to every node of the ANN indicating its impact to other nodes. Figure 2 depicts the architecture of an ANN that consists of an input, a single hidden layer, and an output. In each hidden layer, several computations are performed to determine the input of the next hidden layer. The output $a_{j}^{\left[l\right]}$ of node $z_{j}^{\left[l\right]}$ of the $l^{th}$ hidden layer is defined as:

(3) $$z_{j}^{\left[l\right]}=W^{\left[l\right]}\cdot a^{\left[l-1\right]}+b,$$

(4) $$a_{j}^{\left[l\right]}=f\left(z_{j}^{\left[l\right]}\right),$$

where f, W, and b denote the activation function, weights, and biases. The $z_{j}^{\left[l\right]}$ represents the values computed at each neuron in a particular layer before applying an activation function. These values are the result of linear combinations of the inputs to the neuron, including the weighted sum of input features and a bias term. After computing $z_{j}^{\left[l\right]}$, the next step is to apply an activation function to calculate the output $a_{j}^{\left[l\right]}$. Various activation functions, such as the unit step, rectified linear unit (ReLu) or sigmoid functions could be applied in the $z_{j}^{\left[l\right]}$ values to calculate the output $a_{j}^{\left[l\right]}$. Nonlinear activation functions are widely selected because they introduce complex, nonlinear transformations. A loss function is minimized to approximate the values of the weights. In most regression problems, the Mean Absolute Error (MAE) function is applied, and is defined as:

(5) $$L(y,\;\hat{y_{i}})=\frac{1}{n}*\sum_{i=1}^{n}\left|y_{i}-\hat{y_{i}}\right|,$$

where L(∗), $y_{i}$, and $\hat{y_{i}}$ denote the loss function, the real output, and the output obtained from the *i^th^* layer, respectively [25,26,27]. The backpropagation algorithm minimizes the loss function and is calculated with the following equation:

(6) $$p:=p-a*\frac{\partial L}{\partial p},$$

where p and a denote the learnable parameter and the learning rate of the optimizer, respectively. After training, the ANN model is continuously utilized for decision support. In this context, the ANN model is employed to calculate power predictions based on the available maritime measurements. To select the ideal neural network for calculating predictions, an extended comparison was conducted between ANN and RNN in a previous study [24]. Moreover, Coraddu et al. [16] selected ANNs for predictions concerning propeller-hull degradation. For these reasons, we decided to include the ANN as part of our prognosis method.

### 2.3. Prognosis with Innovative KPI

The estimation of the hull-propeller condition is accomplished through the utilization of an innovative KPI known as m-K3. The DL indicator (m-K3), which was introduced by Theodoropoulos et al. [24], is regarded as an improved version of the international standard ISO-19030 for the purpose of estimating the condition of the hull and propeller. Previous research has demonstrated that the DL indicator can be employed to make more precise and consistent predictions of performance loss over time, in comparison to KPIs based on the ISO-19030. The present study focuses on evaluating the potential of the previously proposed enhanced KPI for forecasting degradation, in addition to detecting it. Mathematically, m-K3 is defined as:

(7) $$m-K3=\frac{P_{measured}-P_{predicted}}{P_{measured}}*100,$$

where $P_{measured}$ and $P_{predicted}$ denote the actual, and the estimated, power measurements of the ship obtained from the ANN model, respectively. The additional filtering is important in order to: (1) avoid numerical errors in the evaluation, and (2) filter out points with bad weather conditions, since the behavior of the vessel in those conditions is strongly inconstant and unreliable. For these reasons, the KPI follows some filtering requirements as described in Part 2 of the ISO-19030 (ISO 2016). Table 1 depicts the filters based on the ISO-19030 that are implemented. The current speed is defined as the difference between the speed over ground and speed through water. The wind speed and current speed parameters refer to the weather conditions, and the filters applied correspond to good weather conditions, following Part 2 of the ISO 19030 (ISO 2016).

In the present study, our objective is to advance the investigation by prognosticating the condition of a vessel’s hull-propeller. This prognostication is accomplished by employing a linear regression model to the KPI values over a designated period, referred to as the hindcast period. The linear regression model can be extrapolated into the future, beyond a specified time point to ascertain the future values of the indicator, which is known as the forecast period. We have opted to employ a simple regression model as it can serve as an effective baseline model. Our prior research has demonstrated that simple regression is a valuable tool for diagnosing the degradation of a vessel’s hull-propeller.

## 3. Data Acquisition

The proposed method is implemented on a large dataset obtained from a LAROS system as presented in detail in [28]. In short, a secure wireless network consisting of smart collectors is set up inside the vessel to transmit the measurements to the gateway at a customizable sampling rate. The wireless network relies on the IEEE 802.15.4 MESH wireless protocols. Additional layers and data formats are applied to cover several requirements of the vessel’s environment and to increase the network’s Quality of Service. The acquired measurements are initially preprocessed, and the data of the collector network are delivered to the gateways. Afterwards, the data are transferred from the gateways to the onboard server. The onboard system periodically creates binary files. The binary files are compressed to reduce the size of the acquired data.

The measurements are obtained at a sampling rate of 1 min and are synchronized using common time stamps. Table 2 presents the main characteristics of the ship under investigation. Table 3 depicts the selected features for implementing the proposed method based on previous works [22,24], and the suggestions of LAROS maritime experts.

**Figure 3:**
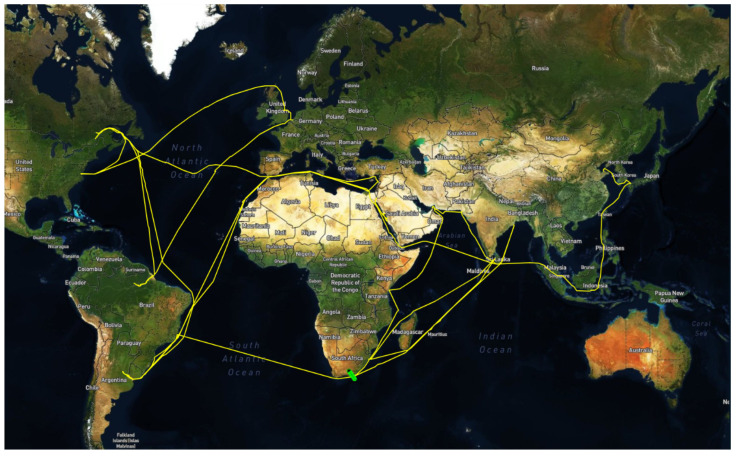
Journeys of the ship under investigation.

**Figure 4:**
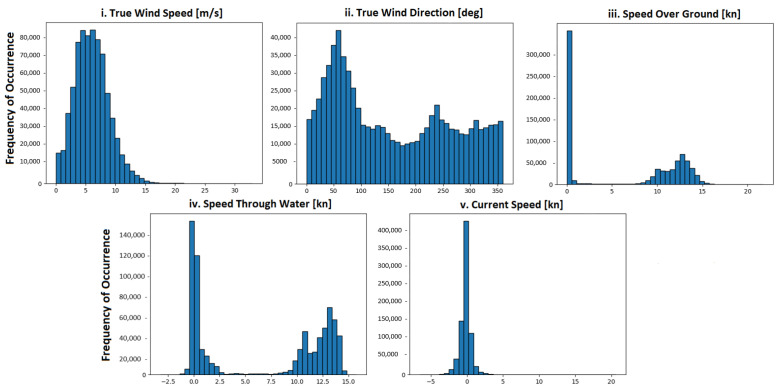
The distributions of: (**i**) true wind speed, (**ii**) true wind direction, (**iii**) speed through water, (**iv**) speed over ground and, (**v**) current speed.

The present dataset comprises measurements pertaining to the operation of a bulk carrier, which were collected over a period of approximately 18 months, spanning from 6 November 2021 to 29 March 2023. Notably, the vessel was new at the onset of the investigation period, which holds significant implications for the proposed methodology. Furthermore, two maintenance actions were carried out on 22 March and 22 August 2022, respectively, namely a propeller and a hull cleaning. Figure 3 depicts the various voyages of the ship, represented by orange lines, while the other colored areas are deemed insignificant for the current study. The dataset exhibits a diverse range of journeys, as evidenced by Figure 3, which is crucial for obtaining generalized outcomes. Figure 4 illustrates the distribution of key parameters, namely true wind speed, true wind direction, speed over ground, speed through water, and current speed, over the 18-month period. The attributes of true wind speed, wind direction, and current speed are associated with weather conditions, and the histograms of all selected features indicate a wide range of conditions. Based on these figures, it can be inferred that the available dataset is a suitable candidate for validating the proposed methodology, given that the collected data are strongly influenced by changes in operational and weather conditions. The dataset’s inherent sensitivity to these real-world factors enhances its utility in assessing adaptability and reliability under diverse, real-world scenarios.

## 4. Results

This section provides a detailed account of the outcomes obtained through the utilization of the proposed DL technique for the prognosis of the propeller-hull condition. The efficacy of the proposed method is assessed through the implementation of diverse experiments.

### 4.1. Preprocessing for ANNs

The initial dataset utilized for the various experiments consists of the measurements outlined in Section 3. This dataset is divided into a training set and a prediction set for each experiment involving ANNs, with the target feature being the propeller shaft power. The training set encompasses measurements taken from 6 November 2021 to 22 March 2022. This training set encompasses a sufficient duration to observe the ship under diverse operational and environmental conditions, without the presence of marine fouling and corrosion. The training period is defined as preceding the first maintenance activity. On the other hand, the prediction set comprises measurements taken from 22 March 2022 to 29 March 2023. The Deep Learning model is trained to model normal system behavior, and to recognize deviations from normality, and thus the training set does not include anomalies and maintenance activities. Testing the model on data containing maintenance activities enables the evaluation of its ability to effectively detect maintenance-related issues and anomalies.

The method presented in this study was evaluated through the implementation of four experiments using ANNs, which will be referred to as ANNs #1–#4 in the following paragraphs. These experiments varied in terms of the selected features, as well as the filters and preprocessing techniques applied to the original dataset. Table 4 provides an overview of the features utilized for each experiment. The strategic use of different feature combinations across datasets in various experiments has the potential to yield better insights and improved model performance. Furthermore, experimenting with different feature combinations enables the identification of the optimal balance between the number of features and model performance to avoid overfitting issues. Table 5 outlines whether the ISO-19030 filters were applied to the training and prediction sets.

**Figure 5:**
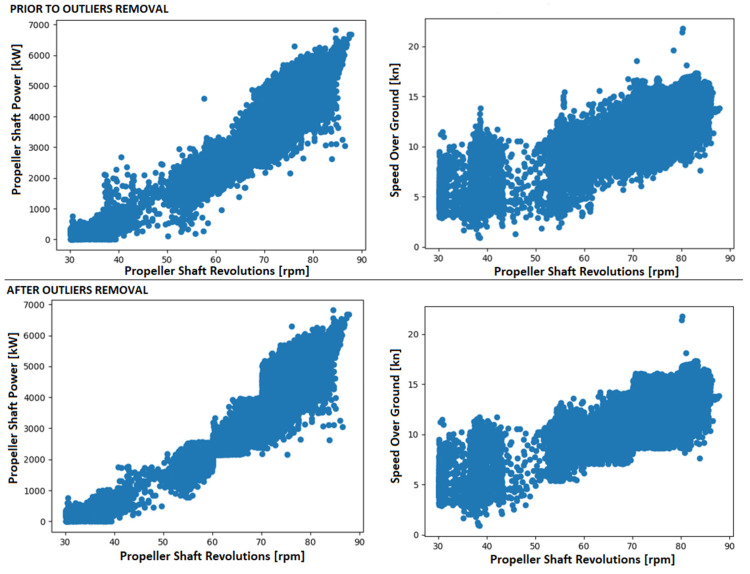
Scatter plots of primary and secondary features prior to and after outliers removal.

**Figure 6:**
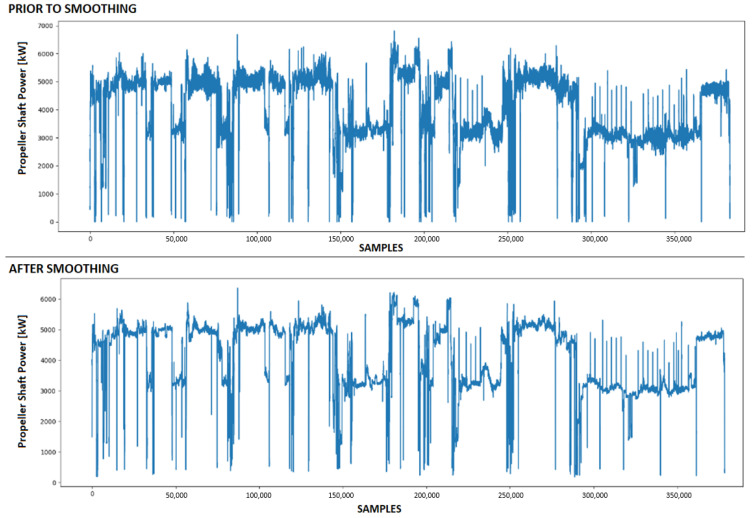
The time-series of propeller shaft power prior to and after smoothing.

In accordance with the preprocessing methods outlined in Section 2, both the outlier technique and the smoothing algorithm were applied to all training and prediction sets. The arbitrary parameter k was set to 3 for the outlier technique, with the propeller shaft revolutions identified as the main feature, while the propeller shaft power and speed over ground were considered secondary features. Figure 5 illustrates the scatter plots of the propeller shaft rpm, propeller shaft power, and speed over ground before and after the removal of the outliers. For the smoothing algorithm, a sliding window of length 5 was utilized, and the results for the propeller shaft power are depicted in Figure 6. The selection of 3 for the outlier technique and 5 for the smoothing algorithm was based on a balance between computational cost and precision, as recommended in [24]. Through experimentation, it was observed that within specific intervals of the propeller shaft rpm, the other two variables exhibited an approximate normal distribution. Consequently, the propeller shaft rpm was designated as the primary feature. As mentioned in [24], values in the tail of the distribution can be eliminated if it is assumed that the second parameter values follow a normal distribution. The values for the preprocessing techniques were determined based on the findings in [24].

In contrast to outlier removal and smoothing techniques, feature selection is exclusively implemented on the training and prediction sets of ANN #2, ANN #3, and ANN #4. Following the application of the Random Forest regressor for feature selection, the parameter pertaining to the commander rudder angle is eliminated from both the training and prediction sets.

### 4.2. Training ANNs

**Figure 7:**
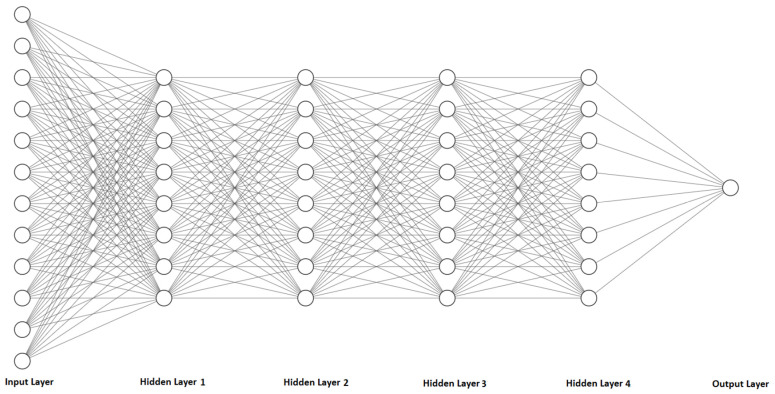
The architecture of the proposed ANN.

**Figure 8:**
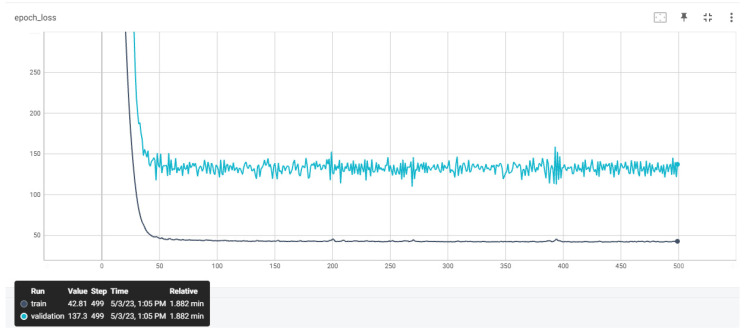
The training and validation loss curves that emerged from ANN #1.

**Figure 9:**
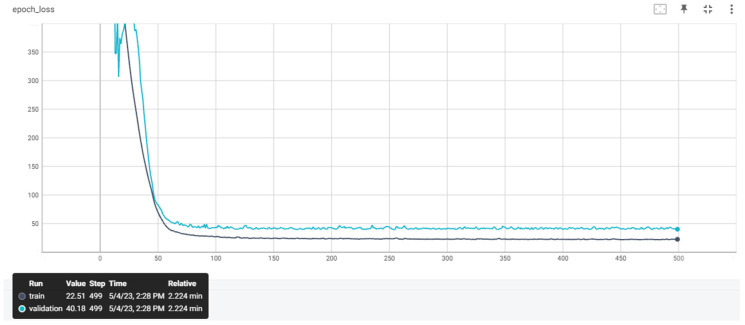
The training and validation loss curves that emerged from ANNs #2 and #3.

**Figure 10:**
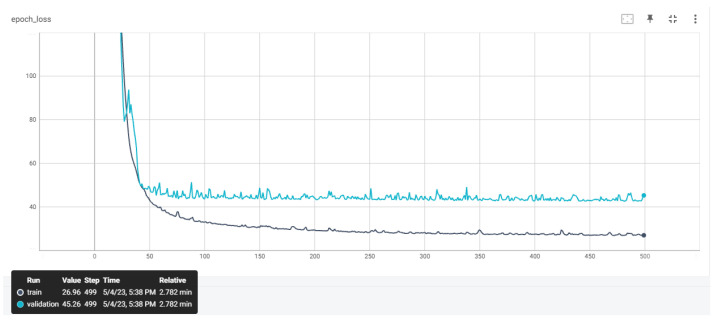
The training and validation loss curves that emerged from ANN #4.

The training datasets previously presented were utilized to train the ANNs. After conducting a thorough investigation into the optimal hyperparameters in our previous studies, we decided to select the same model architecture for the ANNs. Our aim was to examine the impact of different preprocessing techniques on the accuracy of the ANNs with the same architecture. Table 6 provides details of the hyperparameters of the ANNs, including their respective values, while Figure 7 illustrates the architecture of the proposed ANN. Figure 8, Figure 9 and Figure 10 depict the training and validation curves of the four ANNs. The training loss curve indicates the degree to which the model fits the training data, while the validation loss curve indicates the model’s fit to the validation data. ANN #2 and ANN #3 exhibit the same accuracy as the features selected and filters applied in the training set are identical, as indicated in Table 4 and Table 5. The validation loss curve for ANN #2 and ANN #3 converges with the training loss curve, indicating that these models achieve the desired performance rate. Conversely, the validation loss curves for ANN #1 and ANN #4 do not converge with the training loss curves, suggesting that these models are underfitting. Underfitting occurs when a model fails to accurately capture the underlying trend of the data. The preprocessing techniques applied to ANN #2 and ANN #3 prove to be the most effective, as the model architecture remains consistent across all experiments.

### 4.3. Calculating KPI and Forecasting Future Condition

**Figure 11:**
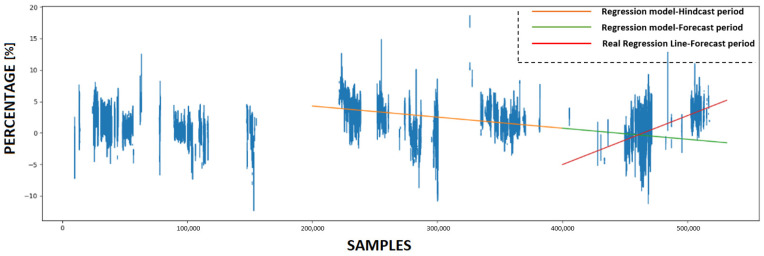
The Key Performance Indicator (KPI) values and the projection of the regression model that emerged from utilizing ANN #1.

**Figure 12:**
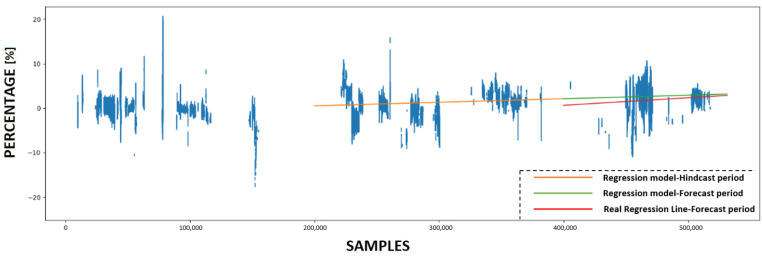
The KPI values and the projection of the regression model that emerged from utilizing ANN#2.

**Figure 13:**
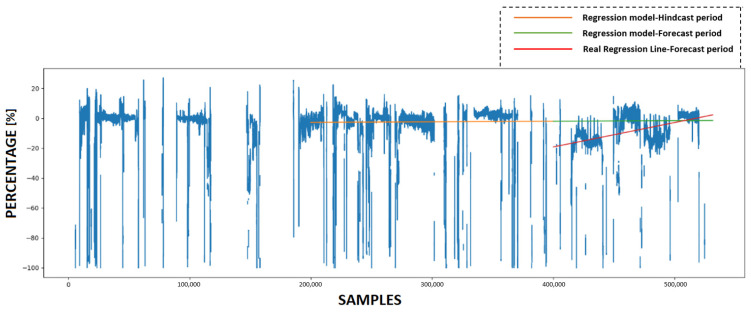
The KPI values and the projection of the regression model that emerged from utilizing ANN #3.

**Figure 14:**
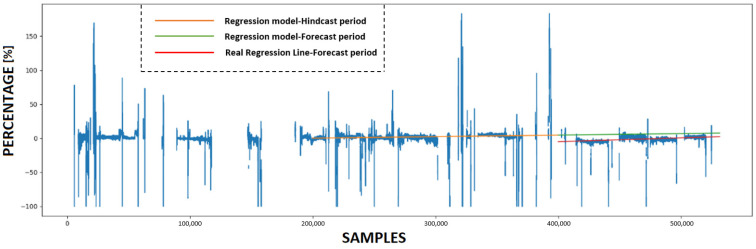
The KPI values and the projection of the regression model that emerged from utilizing ANN #4.

This subsection provides a detailed analysis of the forecasting accuracy of the innovative KPI derived from the use of ANNs. Initially, the trained ANN models utilize various prediction sets to make predictions. Subsequently, four KPIs are calculated based on these predictions, with each experiment having its own KPI. Figure 11, Figure 12, Figure 13 and Figure 14 present the numerical values of the KPIs related to the ship’s propeller-hull performance. In Figure 12, Figure 13 and Figure 14, the method demonstrates expected trends (increasing speed deviation over time). The KPI shown in Figure 12 aligns better with the hull and propeller degradation performance, as the projection qualitatively fits the actual regression line. This suggests that applying similar filters and preprocessing techniques to both the training and prediction sets enhances the accuracy of the method.

In order to assess the predictive capabilities of the proposed methodology, a subset of the available prediction dataset is designated as the hindcast period, while the remaining portion is allocated for the forecast period. In Figure 11, Figure 12, Figure 13 and Figure 14, the initial 200,000 samples following the maintenance activity are utilized as the hindcast period, which spans from 22 August 2022 to 28 December 2022. A linear regression model is then applied to the KPI values during this hindcast period. Subsequently, the regression model is extrapolated into the future for the forecast period, specifically from 28 December 2022 until the conclusion of the prediction period on 29 March 2023. To evaluate the accuracy of this projection, the actual regression line for the forecast period is calculated.

## 5. Comparison with ISO-19030

This section initially presents the baseline method considering propeller-hull degradation, which is based on the ISO-19030. Then, the section compares the prognostic capabilities of the baseline and the proposed method.

### 5.1. Introduction to the Baseline KPI

**Figure 15:**
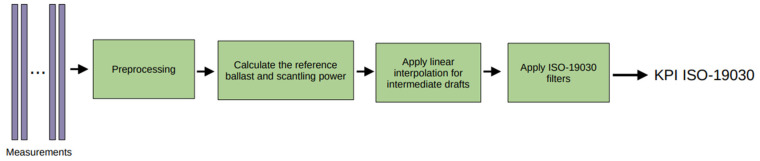
Flowchart to calculate the ISO-19030-based KPI.

This subsection elaborates on the details for calculating the baseline KPI, and Figure 15 illustrates the flowchart for computing it.

**Figure 16:**
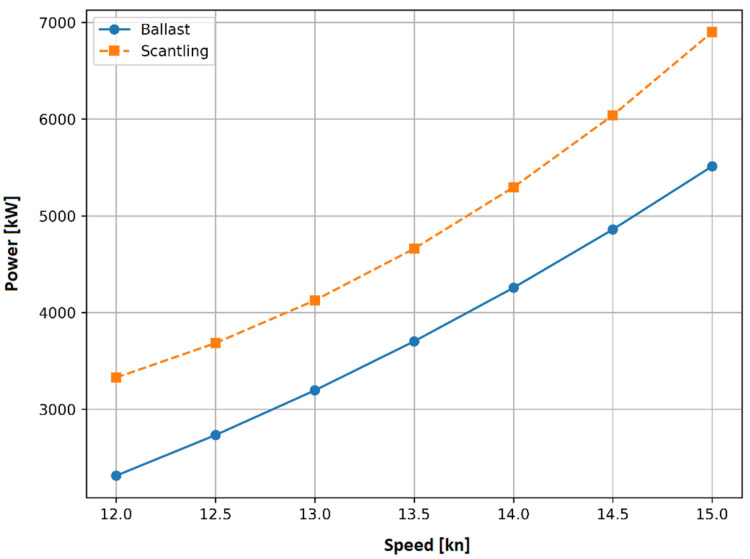
The reference power derived from sea trials for specific speeds, and for ballast (blue) and scantling (orange) conditions.

The computation of the reference power is based on sea trials conducted at specific speeds and load conditions, as depicted in Figure 16. These load conditions encompass a ballast draft and a scantling draft, measuring 5.9 and 13.5 m, respectively. The speed through water varies between 12 and 15 knots for both the ballast and scantling drafts. The reference power for the ballast draft ranges from 2313 to 5512 kW, while for the scantling draft it ranges from 3327 to 6899 kW. In cases where the mean draft falls between the ballast and scantling drafts, the reference power is calculated using linear interpolation.

Additionally, the filters that are listed in Table 1 are applied, and the same preprocessing techniques for removing outliers and smoothing the data are used throughout the analysis. The baseline KPI is computed as the percentage deviation between the measured and the expected-reference propeller shaft power. The ISO-19030-based KPI is mathematically defined as:

(8) $$KPIISO-19030=\frac{P_{measured}-P_{expected}\;}{P_{measured}}*100$$

### 5.2. Comparison between the Proposed and Baseline Method

**Figure 17:**
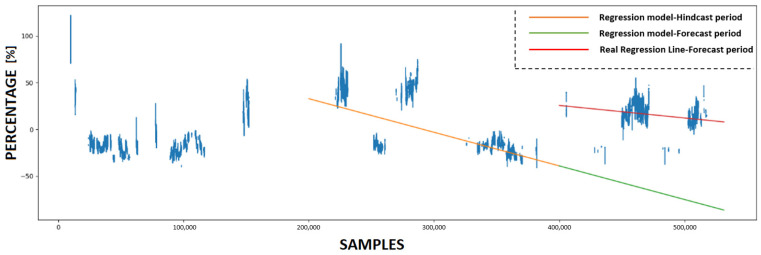
The values of ISO-19030-based KPI and the projection of the regression model.

This subsection presents the results of a comparison between the indicators obtained from four ANNs and the ISO-19030 standard. Figure 17 displays the values of the baseline indicator, while a linear regression model is applied to its values for the same hindcast period as in DL Indicators. The regression model is then projected into the future for the same forecast period, and Table 7 shows the Root Mean Square Error (RMSE) and the MAE between the projection of the regression line and the real regression line for all indicators. According to Table 7, ANN #2 produces the most accurate projection, with the lowest RMSE and MAE values. The DL indicators exhibit higher forecasting accuracy than the baseline indicator in all cases for the vessel under investigation. To verify the results, we applied the proposed method to different types of vessels and calculated the RMSE for ANN#2 and the ISO-19030. Table 8 summarizes the results for a bulk carrier, a container, and an oil tanker. In conclusion, the DL-based method is more efficient for forecasting the degradation of a hull-propeller.

## 6. Conclusions

This paper investigates the implementation of predictive maintenance in ships as a means to mitigate the adverse effects of corrosion and marine fouling, thereby enhancing the efficiency, cost-effectiveness, and sustainability of maritime operations. The primary focus of this study is on predicting the degradation of propeller-hull performance, which poses a significant challenge. The main contributions of this research are twofold: firstly, most existing studies primarily utilize ANNs for diagnosis rather than forecasting propeller-hull degradation, and secondly, a comprehensive comparison is conducted between the DL index and the ISO-19030 KPI using various pre-processing techniques and different types of vessels.

The proposed prognosis method involves initially calculating the DL indicator using ANNs. The prognosis is then achieved by fitting a regression line to the values of the innovative KPI. The DL index demonstrates superior performance, offering valuable insights for future maintenance. It is concluded that selecting a wide range of features and applying consistent filtering and pre-processing techniques enhances the overall performance of the indicator. Conversely, utilizing datasets with fewer features or implementing fewer filters during pre-processing may yield unreliable results. The DL index method can be applied to various maintenance applications beyond the propeller-hull, such as main engines and diesel generators, creating a comprehensive maintenance platform. The utilization of this platform is expected to yield environmental and financial benefits.

The proposed method can be seen as the initial step towards the development of a versatile predictive maintenance system that can diagnose and forecast the degradation of the propeller-hull in various types of vessels. This system would employ transfer learning techniques to extend its applicability across different vessel types, leveraging the knowledge gained from one type to improve performance in another. It is crucial to explore the integration of real-time continuous data streams into the system, and conducting a comprehensive cost-benefit analysis to evaluate the economic advantages of implementing the predictive maintenance system is also recommended. Demonstrating the potential cost savings and reduced downtime associated with predictive maintenance can provide a competitive advantage for the system.

In terms of future research directions, it is recommended that additional pre-processing techniques are explored that can be applied to the measurements in order to obtain the KPIs. Furthermore, it is suggested that the accuracy of advanced regression techniques such as Ridge, Lasso, and Robust regression are investigated. Additionally, the use of hybrid models that combine DL and traditional techniques should be considered to achieve more accurate results.

## Figures and Tables

**Table 1:** Applied filters based on the ISO-19030 standard.

Filters Selected
Wind speed≤7.9 (m/s)
−1≤Current≤1 (knots)
−5≤Commanded rudder angle≤5 (degrees)
12≤Speed through water≤15 (knots)
5.4≤Draft mean≤14 (meters)
51.5≤Propeller shaft revolutions≤88 (rpm)

**Table 2:** Vessel’s main characteristics.

Ship Feature	Value
Ship type	Bulk Carrier
L_BP_	264.00 m
Ballast draft	13.5 m
Scantling draft	5.9 m
Design speed	15 kn
Engine’s Maximum Continuous Rating (MCR)	7300 kW @ 80 RPM
Built in	2020

**Table 3:** Features selected.

Parameter	Units	Measurement Device
Speed over ground	kn	GPS
Speed through water	kn	Speed log
Draft mean	m	Pressure sensor
Propeller shaft revolutions	rpm	Shaft torque meter
Propeller shaft power	kW	Shaft torque meter
True wind speed	m/s	Anemometer
True wind direction	deg	Anemometer
Trim	m	Inclinometer
Vessel heading	deg	Compass
Turbocharger revolutions	rpm	RPM indicator
Scavenger air pressure	bar	Pressure sensor
Commanded rudder angle	deg	Rudder angle
Fuel oil consumption	t/24 h	Mass flow meter

**Table 4:** Features selected for each experiment.

Features	ANN #1	ANN #2, #3, #4
Speed over ground	✓	✓
Speed through water	✓	✓
Draft mean	✓	✓
Propeller shaft revolutions	✓	✓
True wind speed	✓	✓
True wind direction	X	✓
Trim	X	✓
Vessel heading	X	✓
Turbocharger revolutions	X	✓
Scavenger air pressure	X	✓
Commanded rudder angle	✓	✓
Fuel oil consumption	X	✓

**Table 5:** Filters that were applied to the training and the prediction sets.

Dataset	ANN #1 Training	ANN #1 Prediction	ANN #2 Training	ANN #2 Prediction	ANN #3 Training	ANN #3 Prediction	ANN #4 Training	ANN #4 Prediction
Filters	✓	✓	✓	✓	✓	X	X	X

**Table 6:** Hyperparameters selected for ANNs.

Hyperparameter	Value
Learning rate	0.01
Number of hidden layers	4
Number of nodes in each layer	8
Activation function	ReLu
Batch size	4096
Number of epochs	500
Kernel initializer Optimizer	Uniform Adam
Test split	0.2
Shuffle	True

**Table 7:** The RMSE and MAE for all experiments.

KPI Projection Accuracy	RMSE	MAE
ANN #1	3.64	3.150
ANN #2	0.95	0.84
ANN #3	7.4	9.05
ANN #4	7.44	7.55
ISO-19030	79.89	79.43

**Table 8:** The RMSE for ANN#2 and ISO-19030 for different types of vessels.

Vessel	ANN#2	ISO-19030
Bulk carrier	0.95	79.89
Container	8.82	30.24
Oil tanker	3.15	50.23

## Data Availability

Data unavailable—part of a private company’s property.

## References

[1] Oliveira D.R., Granhag L., Larsson L. (2020). A novel indicator for ship hull and propeller performance: Examples from two shipping segments. Ocean Eng..

[2] Tadros M., Ventura M., Guedes Soares C. (2023). Effect of Hull and Propeller Roughness during the Assessment of Ship Fuel Consumption. J. Mar. Sci. Eng..

[3] Guo H.P., Zou Z.J. (2022). CFD and system-based investigation on the turning maneuver of a twin-screw ship considering hull-engine-propeller interaction. Ocean Eng..

[4] Ballou P.J. (2013). Ship energy efficiency management requires a total solution approach. Mar. Technol. Soc. J..

[5] Johnson H., Johansson M., Andersson K., Södahl B. (2013). Will the ship energy efficiency management plan reduce CO_2_ emissions? A comparison with ISO 50001 and the ISM code. Marit. Policy Manag..

[6] Papanikolaou A., Zaraphonitis G., Bitner-Gregersen E., Shigunov V., El Moctar O., Soares C.G., Neddy D.N., Sprenger F. (2016). Energy Efficient Safe SHip Operation (SHOPERA). Transp. Res. Procedia.

[7] Adland R., Cariou P., Jia H., Wolff F.C. (2018). The energy efficiency effects of periodic ship hull cleaning. J. Clean. Prod..

[8] Shaw H.J., Lin C.K. (2021). Marine big data analysis of ships for the energy efficiency changes of the hull and maintenance evaluation based on the ISO 19030 standard. Ocean Eng..

[9] Coraddu A., Figari M., Savio S. (2014). Numerical investigation on ship energy efficiency by Monte Carlo simulation. Proc. Inst. Mech. Eng. Part M J. Eng. Marit. Environ..

[10] Papageorgiou N. (2020). Statistical analysis of marine vessel sensor data using SPM under ISO 19030. Trans Motauto World.

[11] Koboević Ž., Bebić D., Kurtela Ž. (2019). New approach to monitoring hull condition of ships as objective for selecting optimal docking period. Ships Offshore Struct..

[12] Themelis N., Spandonidis C.C., Giordamlis C. (2019). Data acquisition and processing techniques for a novel performance monitoring system based on KPIs. Sustainable Development and Innovations in Marine Technologies, Proceedings of the 18th International Congress of the International Maritime Association of the Mediterranean, (IMAM 2019), Varna, Bulgaria, 9–11 September 2019.

[13] Yu Y., Hoshyar A.N., Samali B., Zhang G., Rashidi M., Mohammadi M. (2023). Corrosion and coating defect assessment of coal handling and preparation plants (CHPP) using an ensemble of deep convolutional neural networks and decision-level data fusion. Neural Comput. Appl..

[14] Yu Y., Li J., Li J., Xia Y., Ding Z., Samali B. (2023). Automated damage diagnosis of concrete jack arch beam using optimized deep stacked autoencoders and multi-sensor fusion. Dev. Built Environ..

[15] Theodoropoulos P., Spandonidis C.C., Fassois S. (2022). Use of Convolutional Neural Networks for vessel performance optimization and safety enhancement. Ocean Eng..

[16] Coraddu A., Oneto L., Baldi F., Cipollini F., Atlar M., Savio S. (2019). Data-driven ship digital twin for estimating the speed loss caused by the marine fouling. Ocean Eng..

[17] Laurie A., Anderlini E., Dietz J., Thomas G. (2021). Machine learning for shaft power prediction and analysis of fouling related performance deterioration. Ocean Eng..

[18] Gupta P., Rasheed A., Steen S. (2022). Ship performance monitoring using machine-learning. Ocean Eng..

[19] Mittendorf M., Nielsen U.D., Bingham H.B. (2023). Capturing the effect of biofouling on ships by incremental machine learning. Appl. Ocean Res..

[20] Uzun D., Demirel Y.K., Coraddu A., Turan O. (2019). Time-dependent biofouling growth model for predicting the effects of biofouling on ship resistance and powering. Ocean Eng..

[21] Nowruzi H. (2022). Performance prediction of stepped planing hulls using experiment and ANNs. Ocean Eng..

[22] Theodoropoulos P., Spandonidis C.C., Themelis N., Giordamlis C., Fassois S. Monitoring of a ship’s energy efficiency based on Artificial Neural Networks and Innovative KPIs. Proceedings of the Annual Meeting of Marine Technology Conference Proceeding.

[23] Christos S.C., Panagiotis T., Christos G. (2020). Combined multi-layered big data and responsible AI techniques for enhanced decision support in Shipping. Proceedings of the International Conference on Decision Aid Sciences and Application.

[24] Theodoropoulos P., Spandonidis C.C., Themelis N., Giordamlis C., Fassois S. (2021). Evaluation of different deep-learning models for the prediction of a ship’s propulsion power. J. Mar. Sci. Eng..

[25] Bhardwaj S., Chandrasekhar E., Padiyar P., Gadre V.M. (2020). A comparative study of wavelet-based ANN and classical techniques for geophysical time-series forecasting. Comput. Geosci..

[26] El-Shafie A., Mukhlisin M., Najah A.A., Taha M.R. (1997). Performance of artificial neural network and regression techniques for rainfall-runoff prediction. Int. J. Phys. Sci..

[27] Obite C.P., Olewuezi N.P., Ugwuanyim G.U., Bartholomew D.C. (2020). Multicollinearity Effect in Regression Analysis: A Feed Forward Artificial Neural Network Approach. Asian J. Probab. Stat..

[28] Christos S.C., Christos G. Data-centric operations in oil & gas industry by the use of 5G mobile networks and industrial Internet of Things (IIoT). Proceedings of the CDT 2018: The Thirteenth International Conference on Digital Telecommunication.

